# Will the real disease gene please stand up?

**DOI:** 10.1186/1471-2156-6-S1-S66

**Published:** 2005-12-30

**Authors:** Neil Shephard, Sally John, Lon Cardon, Mark I McCarthy, Eleftheria Zeggini

**Affiliations:** 1 arc Epidemiology Unit, University of Manchester, Manchester, UK; 2 Centre for Integrated Genomic Medical Research, University of Manchester, Manchester, UK; 3 Wellcome Trust Centre for Human Genetics, University of Oxford, Oxford, UK; 4 Oxford Centre for Diabetes, Endocrinology and Metabolism, University of Oxford, Oxford, UK

## Abstract

A common dilemma arising in linkage studies of complex genetic diseases is the selection of positive signals, their follow-up with association studies and discrimination between true and false positive results. Several strategies for overcoming these issues have been devised. Using the Genetic Analysis Workshop 14 simulated dataset, we aimed to apply different analytical approaches and evaluate their performance in discerning real associations. We considered a) haplotype analyses, b) different methods adjusting for multiple testing, c) replication in a second dataset, and d) exhaustive genotyping of all markers in a sufficiently powered, large sample group. We found that haplotype-based analyses did not substantially improve over single-point analysis, although this may reflect the low levels of linkage disequilibrium simulated in the datasets provided. Multiple testing correction methods were in general found to be over-conservative. Replication of nominally positive results in a second dataset appears to be less stringent, resulting in the follow-up of false positives. Performing a comprehensive assay of all markers in a large, well-powered dataset appears to be the most effective strategy for complex disease gene identification.

## Background

Whole-genome linkage analysis in nuclear families followed by fine mapping studies has been a strategy applied to most common complex genetic traits. LOD scores of genome-wide significance are rarely reached in a single scan due to the inherent lack of power to detect linkage signals for genes of low effect size in commonly used sample sizes. The difficulty in discriminating true signals from type I error is often exacerbated in association studies, where larger numbers of independent tests are carried out in inadequately powered sample sizes. Applying procedures to correct for multiple testing is a robust, but conservative, approach to minimizing type I error. However, for genes with small effect sizes, adjusting for multiple testing in modest sample sizes can lead to an increase in type II error. While the obvious solution is to genotype very large sample sizes for all polymorphisms of interest, this is currently beyond the means of most academic research groups. In addition, there is no standard method for multiple correction that, when applied to haplotype analyses, may increase power to identify disease loci when linkage disequilibrium (LD) exists. An alternative and commonly applied strategy for discriminating between real and false disease loci identified through association studies is to replicate nominally significant results in independent datasets, thereby reducing type II error as all significant results are followed up. Our aims were to use the Genetic Analysis Workshop 14 (GAW14) simulated dataset to carry out linkage analyses and to apply different fine mapping strategies in order to identify true disease loci.

## Methods

### Linkage analysis

Nonparametric linkage analysis was performed on all chromosomes for all 100 replicates in the Aipotu, Karangar, and Danacaa populations using MERLIN (v0.10.1, [1]). Kofendrerd personality disorder (KPD) was the trait used for the whole genome scan. Mendelian inheritance errors were removed prior to analysis using PEDCHECK [2], and MERLIN's error detection algorithm was used to exclude unlikely genotypes prior to analysis. Allele frequencies were estimated from founders in each population. The answers to the simulated dataset were obtained and, in conjunction with the linkage results, 2 true and 2 false positive regions were selected for follow-up, one of each with LD included in the model. Five packets containing genotypes of more densely spaced SNPs were purchased for each region.

### Power calculations

Power calculations were carried out using QUANTO version 0.5.4 [3]. The power attained by using the sample sizes available with each replicate was initially calculated. Subsequently, case-control and family trio sample sizes necessary to attain 50%, 70%, and 80% power to detect effect sizes of odds ratios of 1.3 and 2, for the dominant and recessive models of disease association, were calculated.

### Single-point analysis

The Aipotu and Karangar populations were examined as part of the association analyses. All markers were tested for deviation from Hardy-Weinberg equilibrium (HWE). A case-control sample group and a family trio sample group for transmission-disequilibrium test (TDT) analyses were derived from each replicate. Data for 50 controls per replicate were provided as part of the additional packets that were purchased and 100 cases were derived by selecting one affected individual per family. For each family within each replicate of the Aipotu and Karangar populations 1 trio was derived from the parents and the proband (first affected sibling). Single-point genotypic association was performed by χ^2 ^analysis using STATA v.8 [4]. TDT analyses were also performed, using the TDT routine for STATA written by Clayton (see ). Multiallelic markers with less than 5 transmissions of any allele were excluded from the TDT analysis.

### Multiple testing corrections

Adjusted thresholds for significance of genotypic tests of association and TDT tests were derived based on the family-wise error rate (FWER) correction methods proposed by Bonferroni [5,6], Hochberg [7], and Šidák [8], the false discovery rate (FDR) method proposed by Benjamini and Yekutieli [9] and the *q*-value approach proposed by Storey [10,11].

### Haplotype analysis

LD patterns across the 4 regions of interest were characterized by calculating pairwise LD metrics in population- and replicate-specific cases and controls separately [12]. Average *r*^2 ^values between pairs of adjacent markers were calculated for each region. Haplotype trend regression (HTR), as implemented in HelixTree^®^, was employed to examine disease associations with moving haplotype windows consisting of 2 markers. SNPs deviating from HWE with a *p*-value < 0.001 were excluded from haplotype analyses. In total, 7 SNPs were excluded from haplotype analyses across the 4 replicates because of deviations from HWE. HTR employs the expectation-maximization (EM) algorithm to estimate haplotype frequencies and fits a model of additive effects of haplotypes, using the haplotype probabilities as the regression matrix [13]. TDTPHASE was used to examine deviation from random transmission of haplotypes consisting of 2 contiguous markers [14]. TDTPHASE employs the EM algorithm to infer phase-uncertain haplotypes.

## Results

### Linkage analysis

All 100 replicates were analyzed using nonparametric linkage analysis for the Aipotu, Karangar, and Danacaa populations to localize the true loci. Four regions of linkage were selected for further investigation; 2 true loci on chromosome 3 and 4 and 2 false positive regions both reaching nominal significance in between 8 and 19 replicates on chromosomes 3 and 4 (Table 1). In order to carry out effective haplotype analyses, 1 true and 1 false positive region with simulated LD, according to the description of the simulated dataset, were selected. All subsequent association analyses were carried out in 4 replicates showing evidence of linkage for these loci in the Aipotu and Karangar populations.

### Single-point association analysis

**Figure 1:**
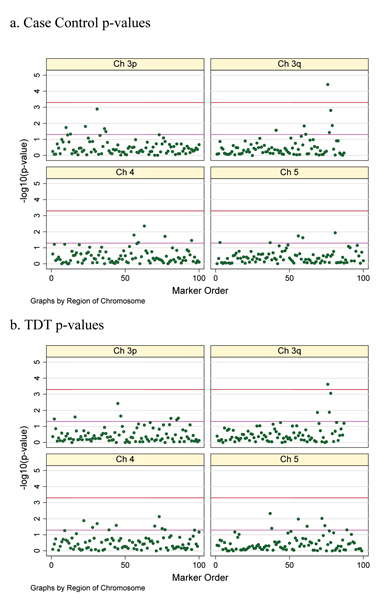
Uncorrected p values for replicate 66 in the Karangar population with a threshold line for the 5% significance level and multiple corrections methods (pink line, *p *= 0.05; red line, *p *= 0.0005 (Bonferroni)).

A case-control set (100 cases and 50 controls) for use in association analysis and a family collection (100 trios) for TDT analysis were defined as described in the methods. Eighty-five markers were analyzed for locus 3_2 and 100 markers for the remaining regions. Markers were associated at *p *< 0.05 for all 4 regions in all 4 replicates when examined using both TDT and case control (approximately 5 positive markers per region per replicate). Figure 1 shows single-point association results for replicate 66, results in the other replicates being comparable. The most significantly associated marker resides on the telomeric end of chromosome 3 (disease locus D2). However, the number of positive results and significance levels at the remaining loci were broadly similar, impeding prioritization for further follow-up. We, therefore, pursued all 4 loci, using the following strategies:

#### Haplotype analysis

Patterns of LD were characterized in cases and controls separately for all markers within each region. Levels of LD were found to be consistently low across regions (in the 4 replicates that were followed up only 4% of adjacent SNPs had an *r*^2 ^> 0.1 across all 4 regions). As the EM algorithm is known to be less robust under low LD in small sample sizes, we restricted multipoint analysis to 2-point haplotypes.

**Figure 2:**
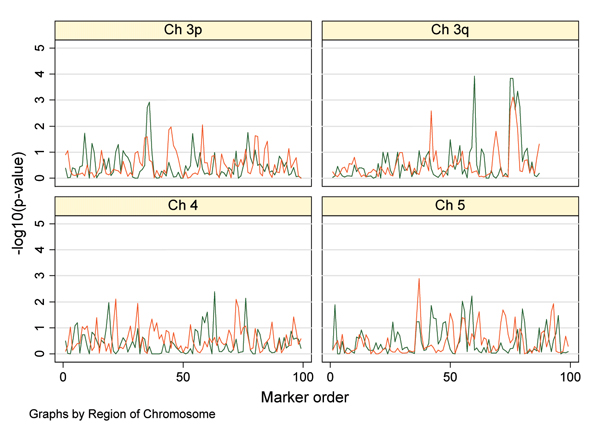
Uncorrected p values from two marker haplotype analysis in replicate 66 in the Karangar population (green line, case-control; orange line, TDT).

Haplotype windows of 2 markers were analyzed in the case-control sample group using HTR. HTR generated significant results in all regions, although the evidence for association was substantially greater in the region containing D2. Similarly, TDT analysis of 2-point haplotypes provided strong evidence for the presence of a true locus at D2. However, it was not possible to differentiate between positive signals at the other loci (Figure 2).

#### Correction for multiple testing

Multiple correction methods controlling the FWER detected D2 on chromosome 3 in 2 out of the 4 replicates analyzed but no other locus remained significant, including D3 (Figure 1).

Different methods for controlling FDR and FWER applied to the data did not make a difference to the interpretation of results. Similarly, different FDR thresholds did not affect the number of *p*-values that remained significant.

#### Replication of results in a larger sample size

A commonly used strategy to overcome the problem of multiple testing is to attempt replication of initial results in a second population. Power calculations showed that the initial sample sizes used had 50% and 69% power for the case-control and TDT analyses, respectively, to detect a genotype relative risk (GRR) of 2 for a dominant model. We calculated the sample sizes required for a GRR of 1.3 as this was the lower limit of the 95% confidence interval calculated for the associated SNP at locus D2. Nine hundred and sixty case-control pairs would be required to detect D3 and 1,079 pairs to detect D2 for a dominant model (80% power at the 5% significance level). Sample groups consisting of 1,000 case-control pairs and 1,000 family trios were constructed from replicates selected randomly for the two populations. These datasets permit evaluation of strategies seeking replication of positive findings, derived from a small initial sample set, in a larger sample size. In addition, examination of these well-powered, exhaustively genotyped datasets facilitates evaluation of comprehensive association study approaches, in which all markers are targeted in sufficient numbers of individuals. When genotype frequency comparisons and TDT analyses were carried out, locus D2 consistently showed strong evidence for association in both populations using both methods. The effect size was estimated as an odds ratio of 2.35 with 95% confidence intervals of 2.07–2.68 (*p *< 1 × 10^-5^). Nominally significant results were found in both populations (*p*-values ranging between 0.05 and 0.01) for the remaining regions. However, the other real disease locus, D3, produced five significant associations at *p *< 0.01. Although evidence for association is not statistically overwhelming for this locus, it would be prioritized above the 2 false positive signals. The effect size for D3 was estimated to have an odds ratio of 1.55 with 95% CIs: 1.35–1.77 (*p *< 10^-5^).

## Discussion

Linkage analysis performed in the Karangar and Aipotu populations generated several peaks of linkage, of which only a proportion harbored susceptibility genes, consistent with findings from many whole-genome screens in complex diseases. We selected four loci demonstrating evidence for linkage in more than one replicate for further investigation, selecting 2 true and 2 false positives. Inspection of the *p*-values generated by single-point analysis in the original sample sizes did not locate the two disease genes with any degree of confidence. Adjusting for multiple testing resulted in the identification of locus D2 only in half of the replicates by both case-control and TDT association analyses, therefore inflating type II error. This was expected, given the gene effect sizes and relatively low power of the sample collections used. The sample sizes used in this study were not atypical of sample sizes reported in genetic association studies. In addition, they were the maximum sizes attainable by using a single replicate. Although the TDT was more powerful in this study, the proportion of positive results was similar to the population-based association analyses carried out.

Different methods to correct for multiple testing were applied to the results, with FWER controlling methods consistently more conservative than FDR controlling ones, although differences were not sufficient to alter the interpretation of results. Adjusting for multiple testing did not facilitate identification of the true disease loci, because in this dataset only one marker exceeded the significance threshold set regardless of the method used. The number of tests carried out in this study amounted to 100 per locus for each replicate.

The functional polymorphism was not genotyped for locus D2. Therefore, we performed haplotype-based analyses with the aim of identifying associated ancestral haplotypes. However, even in regions with simulated LD, the pair-wise LD measures for adjacent markers showed very low inter-marker correlation and, therefore, high haplotype diversity. In reality, haplotype analysis from this type of data would have to be treated with caution, because LD was low and sample sizes small. Two-marker haplotype analysis did, however, provide additional support for the D2 locus, reflecting the results of single-point analyses. The presence of higher LD would be expected to aid in the identification of true loci through multipoint analyses, especially when the relevant functional polymorphisms have not been genotyped.

Given the effect sizes, we constructed datasets large enough to be able to detect the lower confidence limit of the odds ratios by combining sample groups across replicates. This proved to be the most effective strategy to discriminate the location of true disease genes. Disease gene D2 was clearly located with high significance levels in both populations. This is reflected by the larger effect size characterizing this locus. The smaller effect size of D3 hindered its unequivocal identification, demonstrating some of the real challenges of complex disease gene association mapping. The results were equivalent in both populations studied, indicating that the different ascertainment schemes did not qualitatively affect the interpretation of the results.

This study has not taken into account factors such as pathology and underlying biological mechanisms of disease, which could have driven the prioritization of candidate genes within regions and aided the selection of true loci for follow-up. In this context, following a 2-stage approach by seeking replication of significant signals derived from small case-control groups in a larger dataset would result in a trade-off between reducing genotyping effort and identifying true positives. In this study, we would have ultimately followed up more false-positive results and missed D3 in half of the replicates analyzed. Correcting for multiple testing using FWER and FDR controlling procedures can clearly mask true disease genes by being over-conservative, when the sample size is under-powered. Therefore, other correction strategies, such as permutation testing, should be implemented to discriminate between true and false positive signals. Exhaustive genotyping of markers in a well powered dataset of sufficient size proved to be the most effective strategy, ultimately leading to the identification of true disease genes.

## Conclusion

By carrying out sufficiently powered association studies and making use of currently available resources and complementary analytical methodologies, the detection of small genetic effects becomes achievable.

## Abbreviations

EM: Expectation-maximization

FWER: Family wise error rate

FDR: False discovery rate

GRR: Genotype relative risk

HWE: Hardy-Weinberg equilibrium

HTR: Haplotype trend regression

KPD: Kofendrerd Personality Disorder

LD: Linkage disequilibrium

TDT: Transmission-disequilibrium test

## Authors' contributions

NS carried out statistical analyses and helped draft the manuscript. LC and MIM participated in study design. SJ and EZ participated in study design and coordination, carried out statistical analyses and drafted the manuscript. NS, SJ, and EZ also attended the GAW 14 meeting in the Netherlands. All authors have read and approved the final manuscript.

## Figures and Tables

**Table 1:** Summary of regions selected for further fine-mapping analysis.

	Region			
	
	3_1 (3–8 cM)	3_2 (295–300 cM)	4 (14–19 cM)	5 (5–11 cM)
True or false	False	True: D2	False	True: D3
LD	Yes	Yes	No	No
Max NPL score	2.64 (Aipotu rep 40)	4.87 (Aipotu rep 98)	3.35 (Aipotu rep 69)	5.34 (Karangar rep 69)
Replicate 66 (Karangar)	1.75 (*p *= 0.04)	3.32 (*p *= 0.0005)	1.75 (*p *= 0.04)	4.99 (*p *< 0.0001)
Replicate 81 (Karangar)	2.15 (*p *= 0.02)	2.78 (*p *= 0.003)	2.4 (*p *= 0.008)	3.48 (*p *= 0.0002)
Replicate 74 (Aipotu)	1.82 (*p *= 0.03)	2.76 (*p *= 0.003)	3.12 (*p *= 0.0009)	2.53 (*p *= 0.006)
Replicate 88 (Aipotu)	2.08 (*p *= 0.02)	2.41 (*p *= 0.008)	2.2 (*p *= 0.014)	2.41 (*p *= 0.008)
